# Annotations

**Published:** 1891-01-24

**Authors:** 


### Annotations.

Doubtful Cases.

"Drunk or Dying" used to be a frequent heading to paragraphs in professional and other papers, and, unfortunately, errors are still committed in hospitals, though easily preventible. The public are not fully aware how our hospitals are managed so far as the resident medical staff is concerned. In a London Hospital the house surgeon or house physician is usually a senior student recently qualified, who holds the post for six months. In other words, he is young, inexperienced, and impulsive, though generally selected for his marked ability and knowledge of his profession. When a case is brought to the hospital the porter summons one of the residents, who, after an examination, decides whether it shall be admitted or not. Now, as a chain is only as strong as its weakest link, so the reputation of our hospitals mainly hangs upon the absolute discretion of the youngest members of its medical staff. It is time that the medical and house committees looked this question in the face, and that they drew up certain definite instructions with reference to doubtful cases, including the class known as "drunk or dying," and those where the circumstances of the patient make it difficult to determine whether he should be admitted or not. These rules should provide that certain accommodation be set apart in one of the lower rooms or the basement of the institution, where a few bedsteads with straw paliasses and nothing to spoil would make it possible to admit every case of doubt or difficulty, and to retain it until the following morning. In the morning a careful examination could be made and a proper diagnosis arrived at. Until the arrangements here suggested become universal (they are now to be met with in a few of the most intelligently-administered of our hospitals), cases like the recent one at Portsmouth, where the house surgeon refused admission late at night to a man who had broken his leg, because he was very dirty and his clothes contained vermin, will continue to occur. Humanity alone, to say nothing of ordinary discretion, should impel those responsible for the management of every hospital to render it impossible that these cases should occur at all. Now we have shown how easily they may be avoided in future, we hope that a few casual beds of the kind indicated will be provided in every institution.

A French Cure for Consumption.

The activity of medical men all the world over has been stimulated by Dr. Koch's discovery. M. Bertin, of Nantes, and M. Picq have announced a method of treating consumption concerning which the most that can be said at present is that it is novel. So far as is known, the goat is one of the few vertebrate animals that are not liable to tubercular consumption. The two French doctors are of opinion that the cause of the goat's immunity is to be looked for in his blood. They consider it probable that the animal's blood kills the tubercle bacillus. That may be so. If the goat's blood coursing in his own blood vessels destroys the tubercle bacillus, why should not the same blood, transferred to the vessels of a human being, have the same effect? The reasoning has the merit of extreme simplicity. It carefully avoids the ten thousand difficulties that rise up in the mind of the experienced medical man, and takes the shortest possible cut to its goal. M.M. Bertin and Picq have performed the usual experiments. The devoted guinea-pig and the sacrificial rabbit have once more been offered up. Several of these creatures, to whom the physiologists by this time owe many a monument, have been inoculated with tubercule cultures, and have simultaneously had transfused into their circulation a quantity of goat's blood. According to the experimenters, those rabbits inoculated without simultaneous transfusion have invariably died; whilst those transfused at the moment of inoculation have not become tuberculous. Some animals injected first with tubercle and transfused some time afterwards with goat's blood have survived, though tuberculous. These are the justifications for proceeding to treat human beings on the same lines. Accordingly a boy of seventeen and a woman of forty-seven, both suffering from consumption, have each been transfused with about an ounce of goat's blood. The boy's temperature, which had been for several days at 104 deg., a very unusual temperature in phthisis, fell to 98·6 deg., which is the normal temperature of health, and has not risen since. The expectoration is said to have diminished and the mucus to have become non-purulent, whilst the appetite has returned. Now, if these statements be true, particularly that which refers to the temperature, it is not a cure that has been effected, but a miracle which has been wrought. An ounce of goat's blood injected into the body of a grown-up person would not be more than about a two hundredth part of the whole blood circulating in his organism. A most potent remedy, therefore, must goat's blood be if the French doctors are correctly reported. Much more potent indeed than Koch's lymph; and apparently, without any of its dangerous properties. It hardly necessary to say that the story must be taken for what it is worth; and that at the present stage at any rate it is worth exceedingly little. We do not, however, affirm that is worth nothing; nor do we think Koch's failure to establish the validity of his earlier claims should cause a revulsion in the minds of medical men and the public against curative experiments in tuberculosis. On the contrary, it seems highly probable that a method of treating that disease successfully in its early stages will yet be discovered; and that it will be discovered on the lines pursued by the bacteriologists. Some of the most capable scientific workers in this country have for many years been urging their researches in this direction. Being stolid and common sensed Britons, and not ratiocinative Germans; and, having moreover a wholesome horror of quackery before their eyes, they have held their peace until they should have some positive truth to utter. For this, every man of sense and honesty will be grateful to them. Cures let us have by all means, for every disease under the sun; but they must be cures which restore people to health and not cures which send them to their graves.

"We've Got No Work to Do."

The song of the workman as he slowly perambulates the streets where the prosperous dwell is by no means either so tragic or so pathetic as "The Song of the Shirt." But, in so far as the man in fustian and corduroys represents and proclaims an actual fact when he shouts rather than sings that he has "got no work to do," he deserves consideration. Nothing is more demoralising than hunger. Hunger, if it be imperative enough, may by general consent abrogate law. The oldest moralists of whom we have any knowledge taught that a man was hardly to be called a thief who stole to satisfy his hunger. In some Eastern countries not only has hospitality always been recognised as an elementary duty, but a kind of generous carelessness has always been exercised by reapers in order that the poor might glean; and the hungry stranger passing through the fields of standing corn, has had certain prescriptive rights to those ears he could reach with his outstretched hand without departing from the track. The whole history of the world, in fact, shows a certain brotherhood and freemasonry which has always existed between men of all ranks, and of all countries. It has been reserved for London and for the nineteenth century to give birth to the Charity Organisation Society. We do not object to the existence of that society, but we object very strongly to its undue growth. If it is content to remain a frog, and to utter its warning croak only at the proper time, little need be said about it. But it seems inclined to expand into an ox, and to "boss" the whole herd, to use a now familiar Americanism. This can by no means be allowed. Human nature is not going to take either its philosophy or its religion from the Charity Organisation Society. To come to our immediate object: The present weather is bitterly cold: the stomachs of working men, who have no work, must get terribly hungry: the wives and children of such men must experience still keener hunger, because they cannot smoke to keep the fiend away. We counsel all those who have anything to spare to remember the crossing sweeper, the man with his basket of cold and sour oranges, and the belated wearer of "fustian" who has "got no work to do oo-oo." A few shillings thus expended can do the prosperous no harm, and it certainly cannot injure the unprosperous. Hunger has already demoralised them as much as they can be demoralised. Food will put character into them again, and sixpence means a good deal of food to the man who knows how to spend it to the best advantage. It is a great thing we admit to live in the nineteenth century, to be a citizen of London, and a neighbour of the Charity Organisation Society; but it is a much greater thing to belong to all time, to be a freeman of the British Empire, and to be a neighbour of that eastern country where the generous Moses, and One much more generous than Moses lived, and taught, and died. Let us clear ourselves of the cant of a spurious economy.

